# Acute Retinal Necrosis in an Infant With Corneal and Macular Scarring

**DOI:** 10.1177/24741264261430903

**Published:** 2026-04-08

**Authors:** Zuhair Zaidi, Cody Hansen, Sukru Dogan, Angeline L. Wang

**Affiliations:** 1 Department of Ophthalmology, UT Southwestern Medical Center, Dallas, TX, USA; 2 Children’s Health, Dallas, TX, USA

**Keywords:** acute retinal necrosis, neonatal encephalitis, pediatric retina, herpes simplex virus

## Abstract

**Purpose:** To report a rare case of neonatal acute retinal necrosis (ARN) caused by herpes simplex virus type 2 (HSV-2), highlighting its clinical features, diagnostic challenges, and management outcomes. **Methods:** A single case was reviewed. **Results:** ARN is a rare and rapidly progressive ocular condition caused by intraocular herpesvirus infections, including HSV and varicella-zoster virus (VZV). ARN is uncommon in neonates, with few cases documented in the literature. Its nonspecific signs and symptoms, such as redness, eye pain, and decreased vision, combined with the absence of established diagnostic and treatment guidelines, make its recognition in this age group challenging. We report a case of a 33-day-old infant diagnosed with HSV-2–associated ARN, incidentally identified during retinopathy of prematurity screening. The diagnosis was confirmed through characteristic clinical findings and positive serum HSV-2 testing. Prompt antiviral treatment yielded favorable clinical outcomes. Notably, this case demonstrated bilateral macular scars and corneal scarring, both rarely reported in neonatal ARN. **Conclusions:** This case, along with a review of the literature, expands the understanding of ARN in neonates and underscores the importance of early recognition and timely antiviral therapy to optimize visual outcomes.

## Introduction

Acute retinal necrosis (ARN) is a rare and rapidly progressive ocular condition that can affect the retina, uvea, vitreous body, and retinal blood vessels.^1^ ARN is typically unilateral and presents with nonspecific signs and symptoms, including redness, mild ocular pain, and sudden vision loss.^2,3^ It is primarily caused by intraocular herpesvirus infections, such as varicella-zoster virus (VZV), herpes simplex virus types 1 and 2 (HSV-1 and HSV-2), Epstein–Barr virus, or cytomegalovirus.^1–3^ While ARN can occur in individuals of any age or sex, it is uncommon in infants and neonates, with only a limited number of cases documented in the literature.^4–14^ Currently, no established guidelines exist for the diagnosis and treatment of ARN in this age group. Recognizing ARN in neonates and infants can be challenging owing to its rarity and nonspecific signs and symptoms. Additionally, approximately 70% of affected neonates are born to asymptomatic mothers, further complicating timely diagnosis.^15^

This case report sheds light on the clinical course, treatment, and outcome of an infant diagnosed with ARN and provides a comprehensive review of the existing literature on ARN associated with infantile and neonatal HSV infections.

A retrospective chart review of an infant diagnosed with ARN caused by HSV-2 infection was performed. For the literature review, we conducted a systematic search of PubMed/MEDLINE, Embase, and Scopus from January 1980 through February 2025. This time frame was chosen to capture all published cases since the first descriptions of ARN in the 1980s. Search terms included “acute retinal necrosis,” “ARN,” “neonatal acute retinal necrosis,” “infant ARN,” “herpes simplex virus retinitis,” and “pediatric ARN,” combined using appropriate Boolean operators. Reference lists of relevant articles were also manually reviewed to ensure completeness.

Inclusion criteria were: (1) case reports, case series, meta-analyses, or reviews describing ARN in neonates (≤28 days) or infants (<1 year); (2) confirmed diagnosis of ARN based on characteristic clinical findings and/or laboratory confirmation of viral etiology; and (3) sufficient clinical detail to allow abstraction of patient-level data.

Exclusion criteria were: (1) studies that did not involve infants or neonates; (2) studies without individual-level data (eg, narrative reviews without case-specific detail); and (3) duplicate reports of the same patient. To minimize double counting, we cross-checked authors, institutions, patient demographics, and clinical details across case reports, case series, and meta-analyses. When overlap was suspected, only the most detailed or original report was included.

From each eligible study, we extracted data on patient demographics, viral etiology, clinical presentation, diagnostic methods (serum, cerebrospinal fluid [CSF], aqueous/vitreous samples, skin testing, or imaging), treatment regimen (systemic, intravitreal, laser, or surgical), complications (eg, retinal detachment, optic atrophy, corneal involvement), and clinical outcomes.

## Case Report

**Figure 1.**
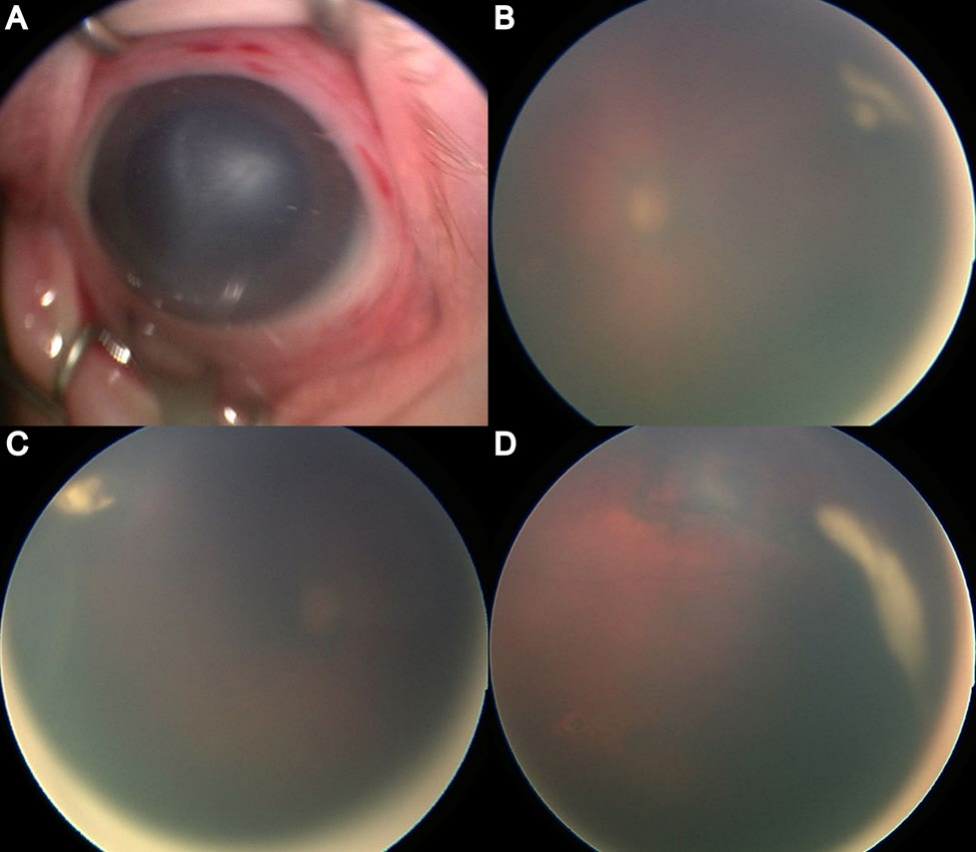
(A) Anterior segment photograph demonstrates corneal stromal haze and conjunctival injection. (B–D) Widefield fundus images of both eyes show dense vitritis limiting visualization of the posterior pole, with faintly visible peripheral whitish retinal lesions consistent with necrotizing retinitis.

A 33-day-old male infant, born at 30 weeks and 4 days of gestation with a birth weight of 1540 g, was found to have vitritis, bilateral white retinal lesions, and anterior corneal stromal haze in the right eye during a routine retinopathy of prematurity screening. Visualization of the retina was limited owing to dense vitritis; however, peripheral white lesions were identified in both eyes, along with bilateral retinal pigment epithelium (RPE) mottling and a hemorrhage along the superior macula of the left eye. The medical history of the patient was notable for neonatal sepsis, grade III intraventricular hemorrhage, bronchopulmonary dysplasia, anemia of prematurity, and patent ductus arteriosus, while the maternal history was significant for treated gonorrhea infection during pregnancy, prolonged rupture of membranes, and placental chorioamnionitis. Maternal testing for syphilis, HSV, human immunodeficiency virus, and group B *Streptococcus* was negative. The neonate had previously received 3 weeks of cefepime for presumed meningitis; however, initial CSF cultures were negative. Figure 1 depicts initial examination findings.

Examination findings were highly concerning for infectious chorioretinitis. Laboratory evaluation revealed a positive serum HSV-2 polymerase chain reaction (PCR). Lumbar puncture revealed low glucose, elevated protein levels, and high nucleated cell count. Magnetic resonance imaging of the brain revealed evidence of remote injury involving the left posterior frontal lobe, with cystic encephalomalacia and Wallerian degeneration of the left cerebral peduncle. Additional findings included sequelae of prior hemorrhage in the bilateral thalami and encephalomalacia of the posterior putamina. Notably, aqueous or vitreous sampling was not performed owing to concerns regarding the safety of a bedside procedure and the risks associated with general anesthesia in this medically fragile infant.

**Figure 2.**
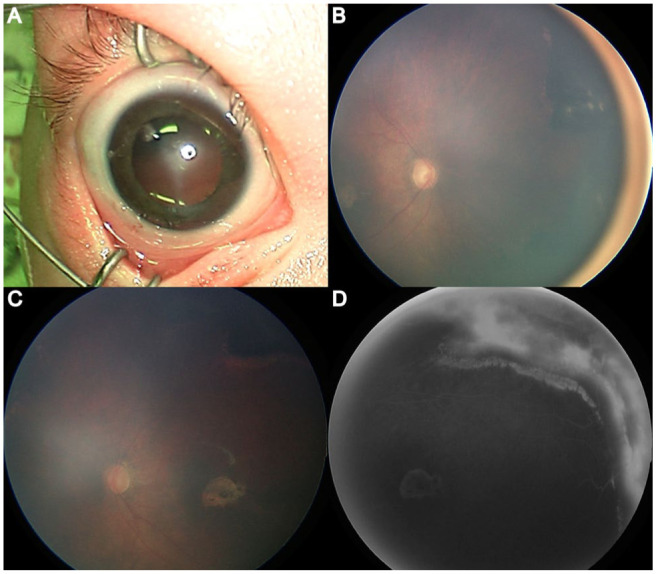
(A) Anterior segment photograph demonstrates persistent corneal stromal haze. (B) Widefield color fundus image shows improved media clarity with visualization of the optic nerve and posterior pole following partial resolution of vitritis. (C) Peripheral fundus photograph reveals areas of necrotizing retinitis with evolving retinal whitening and early scarring. (D) Widefield fluorescein angiography image demonstrates peripheral nonperfusion and staining corresponding to areas of necrotizing retinitis.

The patient was initially treated with intravenous acyclovir (20 mg/kg/dose every 8 hours) for 28 days, as recommended by the infectious disease team. Bilateral intravitreal foscarnet injections (0.72 mg in 0.03 mL per eye) were also administered. Over the next 6 weeks, the vitritis and retinitis slowly resolved, revealing 360-degree peripheral retinal scarring and pigmentary changes that were more extensive than initially visible. Bilateral macular scars also became evident. The corneal haze gradually improved over the same period. Figure 2 depicts updated examination findings.

The patient was subsequently transitioned to oral acyclovir (300 mg/m^2^ per dose, 3 times daily) for 1 year. After the infection resolved, prophylactic laser retinopexy was performed to reduce the risk of retinal detachment. At the most recent follow-up, 24 months after the initial diagnosis, the retinal examination was stable, with no recurrence of retinitis or evidence of retinal detachment. The corneal stromal haze had also improved significantly.

Notably, the patient was re-admitted about 9 months after the initial infection owing to the development of a vesicular rash on the hands and feet along with positive HSV-2 blood cultures consistent with disseminated HSV-2 infection. This episode was thought to be related to systemic corticosteroid therapy that had been started for treatment of infantile spasms. Importantly, there was no recurrence of ARN during this episode. The patient was successfully treated with intravenous acyclovir.

## Conclusions

Neonatal HSV infection, occurring in approximately 1 in 3000 live births per year in the United States, primarily results from vertical transmission from mothers with genital HSV infections.^16^ During the perinatal period, the virus gains access to the neonate through sensory nerves, such as those found in the conjunctiva or nasal epithelium, subsequently traversing sensory neural pathways, including the olfactory and trigeminal nerves, leading to primary infection. Clinical manifestations of neonatal HSV infection span from localized skin, eye, and oral involvement to disseminated infection, often accompanied by neurologic complications such as encephalitis and seizures. Untreated disseminated or neurologic cases carry a mortality rate of up to 80%.^17–19^ ARN is a severe ocular complication of neonatal HSV infection, typically occurring in advanced stages of the disease and strongly associated with vision-threatening complications such as retinal detachment and subsequent blindness.^20,21^

Our literature review through February 2025 identified 18 reports of neonates and infants with ARN.^4–14^ We analyzed data from 19 patients (34 eyes), including the patient treated at our center. The demographic information, clinical characteristics, laboratory results, treatment approaches, and outcomes of these cases are presented in Table 1, with detailed information about each individual case provided in the Supplemental table. The majority of patients reported in the literature had bilateral involvement (15/19, 79.0%), with most testing positive for HSV-2 (84.2%). Diagnosis was confirmed using a variety of methods, including CSF analysis, serum testing, skin lesion sampling, and aqueous or vitreous fluid analysis.

**Table 1.** Summary of Published Cases of Neonatal ARN.

Demographics	Patients / Eyes	19 / 34
	Male / Female	11 (57.9%) / 8 (42.1%)
	Bilateral	15 (79.0%)
	Right eye only / Left eye only	2 (10.5%) / 2 (10.5%)
	HSV-1	3 (15.8%)
	HSV-2	16 (84.2%)
Clinical presentation	Neurologic complications^a^	11 (57.9%)
	Seizures	7 (36.8%)
	Rash	7 (36.8%)
	Found incidentally	6 (31.6%)
	Apneic spells	3 (15.8%)
	Lethargy	2 (10.5%)
	Fever	1 (5.3%)
Comorbidities	Premature birth	12 (63.2%)
	Respiratory distress syndrome	5 (26.3%)
	Retinopathy of prematurity	5 (26.3%)
	Patent ductus arteriosus	2 (10.5%)
	Intraventricular hemorrhage	2 (10.5%)
	Atrial septal defect	1 (5.3%)
	Candidemia	1 (5.3%)
	Necrotizing ileitis	1 (5.3%)
	Sepsis	1 (5.3%)
Ocular presentation	Anisocoria	3 (15.8%)
	Poor vision	1 (5.3%)
	Redness	1 (5.3%)
Diagnostic methods	CSF PCR	7 (36.8%)
	Serum IgM/IgG	5 (26.3%)
	Aqueous PCR	5 (26.3%)
	Serum PCR	4 (21.1%)
	Skin lesion PCR	2 (10.5%)
	Vitreous PCR	2 (10.5%)
	Vitreous IgM/IgG	1 (5.3%)
	Throat swab culture	1 (5.3%)
	Skin lesion culture	1 (5.3%)
	Conjunctival swab immunofluorescent antibody test	1 (5.3%)
	CSF anti-HSV antibody, viral neutralization test	1 (5.3%)
Ocular complications	Retinitis, location periphery	22 (64.7%)
	Vitritis	14 (41.2%)
	Retinitis, location posterior	10 (29.4%)
	Optic atrophy or disc edema	7 (20.6%)
	RD (type tractional), vitreoretinal traction, PVR	6 (17.6%)
	RD, type not specified	4 (11.8%)
	Corneal edema, haze, scarring	4 (11.8%)
	Cataracts, lens changes	4 (11.8%)
	Subretinal hemorrhage	3 (8.8%)
	RD, type rhegmatogenous	3 (8.8%)
	RD, type exudative	3 (8.8%)
	Vitreous hemorrhage	3 (8.8%)
	Previous chorioretinal scarring	3 (8.8%)
	Macular scarring or macular RPE changes	3 (8.8%)
	Macular exudates	3 (8.8%)
	Anterior chamber inflammation	3 (8.8%)
	Retinitis (location not specified)	2 (5.9%)
Treatment	Intravenous acyclovir (treatment dose)	18 (94.7%)
	Oral acyclovir/valacyclovir (maintenance dose)	12 (63.1%)
	Topical steroids	5 (26.3%)
	Systemic steroids	2 (10.5%)
	Intravitreal antivirals	2 (10.5%)
	Oral acyclovir (treatment dose)	1 (5.2%)
Surgical intervention	Laser retinopexy	11 (32.4%)
	Pars plana vitrectomy	3 (8.8%)
	Scleral buckle	2 (5.9%)

Abbreviations: ARN, acute retinal necrosis; CSF, cerebrospinal fluid; HSV, herpes simplex virus; Ig, immunoglobulin; PCR, polymerase chain reaction; PVR, proliferative vitreoretinopathy; RD, retinal detachment; RPE, retinal pigment epithelium.

a Neurologic complications encompass encephalitis, encephalomalacia, intracranial hemorrhage, and periventricular leukomalacia.

At presentation, clinical features suggestive of HSV infection varied. Neurologic complications, including encephalitis, encephalomalacia, intracranial hemorrhage, and periventricular leukomalacia, were observed in 11 patients (57.9%), while seizures and rash were each reported in 7 patients (36.8%). In 6 cases (31.6%), ARN was discovered incidentally in the absence of notable systemic symptoms. Among those with ocular symptoms, anisocoria was reported in 3 patients (15.8%), while decreased vision and redness were each documented in 1 patient (5.3%).

The most commonly reported ocular examination findings were peripheral retinitis in 24 eyes (70.6%), vitritis in 12 eyes (35.3%), and posterior retinitis in 10 eyes (29.4%). Retinal detachment was a frequent complication, including 6 tractional, 3 rhegmatogenous, and 3 exudative detachments, with 4 additional cases in which the type was not specified. Optic atrophy or optic nerve edema was reported in 7 eyes (20.6%).

Notably, our case represents the sole documented case of bilateral macular scarring secondary to neonatal ARN in the literature and involves 1 of 3 reported patients with corneal haze or scarring.^5,6,8^ In our patient, the corneal findings were likely attributable to concomitant herpetic keratitis. A case series of adult patients demonstrated that 5 out of 28 patients who developed ARN due to HSV also had concomitant keratitis; among these, 3 were diagnosed with disciform keratitis and 2 with stromal keratitis.^22^

Universal neonatal eye screening, whether conducted in person or via telemedicine using widefield imaging, has gained increasing recognition as a method to detect significant ocular disease far earlier than routine pediatric examinations. Although no formal guidelines from major pediatric ophthalmology societies specifically mandate eye examinations for infants born to mothers with HSV-2 infection, multidisciplinary clinical pathways address this concern. The Johns Hopkins Neonatal Intensive Care Unit clinical pathway (June 2025) recommends that infants born to mothers with known or suspected HSV infection undergo routine ophthalmologic examination as part of their early evaluation, even in the absence of ocular symptoms.^23^Similarly, the Canadian Paediatric Society advises that infants with suspected or confirmed neonatal HSV infection be referred for ophthalmology consultation to assess for potential ocular involvement.^24^

While birth-related retinal hemorrhages remain the most common finding during neonatal eye screenings, occurring in approximately 13% to 30% of screened infants, studies consistently demonstrate that 2% to 3% of newborns harbor serious abnormalities requiring intervention, including congenital cataracts, glaucoma, retinoblastoma, or retinal infections.^25–27^ For instance, a national screening program in Turkey reported that, of more than 1000 screened infants, 0.67% required surgical or chemotherapeutic intervention.^28^

In our review of neonatal HSV-associated ARN, 63% of infants had central nervous system involvement, 31% were diagnosed incidentally, and approximately 70% were born to asymptomatic mothers, highlighting the difficulty of clinical recognition without ophthalmic screening.^4–14^ Our patient was diagnosed at 33 days of life solely because of a retinopathy of prematurity screening examination, highlighting how early detection can help prevent downstream sequelae such as retinal detachment—documented in up to 80% of delayed cases—and neurologic injury from untreated HSV encephalitis.^29^

Together, these findings support universal neonatal eye screening as a feasible strategy to mitigate irreversible visual and systemic morbidity.

The treatment approach for ARN in adults, as outlined by the American Academy of Ophthalmology, generally begins with a 5- to 10-day course of intravenous acyclovir at a dose of 10 mg/kg every 8 hours, followed by transition to oral antiviral therapy.^30–33^ In children, however, well-established therapeutic guidelines remain lacking.

According to our literature review, intravenous acyclovir was the primary antiviral treatment, administered in 18 cases (94.7%) at doses ranging from 10 mg/kg to 20 mg/kg 3 times daily for an average duration of 3 to 4 weeks. For long-term antiviral prophylaxis, 12 patients (68.4%) were transitioned to oral acyclovir or valacyclovir, with treatment durations extending up to 4 years in some cases. Systemic steroids to reduce inflammation were administered in 2 cases (10.5%), and topical steroids were used in 5 cases (26.3%).

In the adult literature, intravitreal injections of ganciclovir and foscarnet are well-established for delivering immediate, high-dose treatment directly to the affected eye.^33,34^ Among the reported neonatal ARN cases, 2 patients, including the present case, received intravitreal injections of foscarnet. No adverse side effects associated with these treatments were reported in these cases.

In total, 7 patients (11 eyes), including our patient, underwent prophylactic laser retinopexy. Among these 11 eyes, 1 subsequently developed a rhegmatogenous retinal detachment and another developed a tractional detachment, both of which were successfully repaired with scleral buckle surgery. A 2022 meta-analysis of 14 studies and 532 eyes by Chen et al^35^ demonstrated that prophylactic laser reduced the risk of retinal detachment by 39%. The risk was further reduced in patients treated with a combination of antiviral therapy and corticosteroids, suggesting that more rapid resolution of retinitis and inflammation may improve the efficacy of laser retinopexy.

Given that retinal detachment repair can be particularly challenging in neonates and young infants, we recommend consideration of prophylactic laser retinopexy for neonatal patients with ARN with high-risk presentations. Despite extensive peripheral retinitis, our patient remained free of retinal detachment at the 24-month follow-up.

The visual prognosis for patients with ARN is generally poor, as indicated by both the pediatric and adult literature.^36–40^ In a study by Iwahashi-Shima et al,^41^ which included 104 patients aged 12 to 79 years with ARN, 56 eyes (53.8%) had a best-corrected visual acuity (BCVA) of 20/200 or worse after 1 year. The study further detailed that BCVA improved by more than 3 lines in 23 patients, remained stable in 34 patients, and deteriorated by more than 3 lines in 47 patients.

In our review of neonatal cases, the follow-up period did not extend to ages at which visual acuity could be reliably assessed. In older patients, several factors, including advanced disease at presentation, the occurrence of retinal detachment, and older age at onset, have been associated with worse visual outcomes.^42–45^

In conclusion, isolated ARN in neonates and infants remains an infrequent yet critical clinical entity. While 63% of patients in our literature review were found to have signs of viral encephalitis—such as seizures or spasms—that prompted neurologic examination, 31% did not present with systemic symptoms, and ARN was discovered incidentally. Additionally, approximately 70% of affected neonates are born to asymptomatic mothers, further emphasizing the importance of vigilance among clinicians.^36^

When evaluating neonates with ocular manifestations suggestive of ARN, immediate diagnostic testing for HSV and other TORCH infections, along with empiric initiation of intravenous acyclovir, is essential. Our case further demonstrates that concomitant stromal keratitis may occur in patients with ARN and that long-term sequelae can include macular scarring. These distinctive findings underscore the diverse clinical presentation of neonatal ARN and reinforce the importance of early diagnosis and proactive management, particularly given the substantial risks associated with HSV encephalitis in this vulnerable population.

## Supplemental Material

sj-docx-1-vrd-10.1177_24741264261430903 – Supplemental material for Acute Retinal Necrosis in an Infant With Corneal and Macular Scarring Supplemental material, sj-docx-1-vrd-10.1177_24741264261430903 for Acute Retinal Necrosis in an Infant With Corneal and Macular Scarring by Zuhair Zaidi, Cody Hansen, Sukru Dogan and Angeline L. Wang in Journal of VitreoRetinal Diseases
