# Supplementary material for: Acute Retinal Necrosis in an Infant With Corneal and Macular Scarring
Source: J Vitreoretin Dis. 2026 Apr 8:24741264261430903. Online ahead of print. doi: 10.1177/24741264261430903 (PMC13061849; doi:10.1177/24741264261430903)
Supplement: sj-docx-1-vrd-10.1177_24741264261430903 – Supplemental material for Acute Retinal Necrosis in an Infant With Corneal and Macular Scarring [file sj-docx-1-vrd-10.1177_24741264261430903.docx]

| Reference | Age | Sex | Eyes | Laterality | Virus | Diagnostic Method | Maternal History | Comorbidities | Systemic Presentation | Ocular Signs | Eye Exam Findings | Antiviral Treatment | Steroids | Surgery | Follow-Up |
| --- | --- | --- | --- | --- | --- | --- | --- | --- | --- | --- | --- | --- | --- | --- | --- |
| Kychenthal et al., 2001 | 27d | M | 2 | OU | HSV2 | Skin lesion PCR, CSF PCR, throat swab culture | Positive titers in third trimester | - | Lethargy, cough, poor feeding, rash, encephalitis | - | Peripheral retinitis OU → RD OU, large subretinal hemorrhage OS | IV(Ac) + O(Ac) | - | Laser OD, PPV OS | - |
| Gupta et al., 2010 * | 25d | M | 2 | OU | HSV2 | Conjunctival swab immunofluorescent antibody, serum IgM | Genital lesions, positive IgM and negative IgG | Preterm (33w), twin | Lethargy, weight loss, rash, encephalitis | Redness OD | Corneal stromal edema OD, conjunctival congestion OD, posterior retinitis OU → optic atrophy OS, RRD OS | IV(Ac) 20mg TID x 21d + O(Ac) 40mg BID x 6w | Fluoromethalone gtts | - | 3m |
| Gupta et al., 2010 * | 25d | M | 2 | OU | HSV2 | - | Genital lesions, positive IgM and negative IgG | Preterm (33w), twin | Encephalitis, apneic spells | - | Peripheral retinitis OU | IV(Ac) | - | - | Died 5d after diagnosis |
| Silva et al., 2013 | <1y | F | 1 | OD | HSV2 | Serum PCR | - | Atrial septal defect | Seizures | Anisocoria OD | Peripheral retinitis OD → RD OD | IV(Ac) x 0.9m | Prednisolone gtts | - | - |
| Silva et al., 2013 | <1y | F | 1 | OD | HSV2 | Aqueous PCR | Positive serum PCR | - | Neonatal HSV, seizures, metabolic acidosis | Anisocoria OD | Retinitis OD, CRS OD → PVR OD | IV(Ac) x 0.7m | Prednisolone gtts, oral prednisone | - | - |
| Silva et al., 2013 | <1y | F | 1 | OS | HSV2 | Serum IgM/IgG, vitreous IgM/IgG | Genital lesions | - | Neonatal HSV, encephalomalacia | Anisocoria OS | Peripheral retinitis OS → RD OS | IV(Ac) x 0.1m + O(Ac) x 22.8m | - | Laser OS | - |
| Hsu et al., 2013 | 1d | M | 2 | OU | HSV2 | Aqueous PCR, serum IgG | PPROM, positive serum IgG and negative serum IgM | Preterm (29w), di-di twin, IVF with donor egg | Rash | - | Vitritis OU, peripheral retinitis OU → RRD in one eye, macular pigment changes, optic atrophy | IV(Ac) 10mg/kg TID x 5-10d + O(Ac) 800 mg 5x/d x 6w | - | Laser OU, SB in one eye | 6m |
| Fong et al., 2014 | 26d | F | 2 | OU | HSV1 | CSF PCR, vitreous PCR | Positive serum IgM and negative IgG | - | Focal seizures, encephalitis | - | Non-specific posterior exudates OS → vitritis OS, optic nerve swelling OS, peripheral retinitis OS → peripheral retinitis OD, TRD OS | IV(Ac) 20mg/kg TID x 5w + O(Ac) x 6m + weekly intravitreal foscarnet (1.2mg) OU x4 | Oral steroid 1 mg/kg/day x 1m | PPV OS | 6m |
| Venincasa et al., 2015 | 2m (48 days) | F | 2 | OU | HSV2 | Aqueous PCR | - | Preterm (32w) | Seizures, intracranial hemorrhage, periventricular leukomalacia, diffuse encephalopathy | - | Vitritis OD, exudative RD OD, peripheral retinitis OD → retinitis OS | IV(Ac) x 3w | Prednisolone gtts |  | - |
| Venincasa et al., 2015 | 2m (48 days) | F | 1 | OS | HSV2 | Serum titers, serum PCR | - | - | Seizures, intracranial calcifications | Poor vision OS | Vitreoretinal traction OS, peripheral retinitis OS, vitreous hemorrhage OS | O(Ac) | - | Laser OS | - |
| Venincasa et al., 2015 | 5w (35 days) | M | 2 | OU | HSV2 | Aqueous PCR | - | Preterm (29w), twin, ROP | Rash | - | Vitritis OU, peripheral retinitis OU, corneal haze OU → TRD OD, corneal scar OS | IV(Ac) + O(Ac) x 8w | - | Laser OU, SB OD | - |
| LaMattina et al., 2016 | 5w (35 days) | M | 2 | OU | HSV2 | CSF PCR | - | Preterm (27w), ROP, RDS | - | - | Peripheral cataracts OU, peripheral retinitis OU | IV(Ac) 20 mg/kg TID x 3w + O(Ac) 20mg/kg BID x 4y | - | Laser OU | 4y |
| LaMattina el al., 2016 | 3w (21 days) | F | 2 | OU | HSV2 | Skin lesion culture, CSF PCR | PPROM | Preterm (26w), ROP, RDS | Rash | - | Posterior retinitis OU → traction | IV(Ac) 20 mg/kg TID x 3w + O(Ac) 20 mg/kg BID x 4y | - | - | 4y |
| Ren et al., 2019 | 23d | M | 2 | OU | HSV2 | Serum PCR, CSF PCR | - | - | Fever, seizures, bulging fontanelle, nuchal rigidity | - | Peripheral retinitis OU | IV(Ac) x 2m + O(Ac) x 6m | - | - | 6m |
| Khurram et al., 2020 | 10w (70 days) | M | 2 | OU | HSV1 | Skin lesion PCR, aqueous PCR, vitreous PCR | Vaginal swab positive for candida | Preterm (24w), ROP, RDS, candidemia, IVH, necrotizing ileitis | Rash, apneic spells |  | Rubeosis and anterior chamber fibrin OU, vitreous hemorrhage OU, posterior retinitis with subretinal hemorrhage OU | IV(Ac) 2 mg/kg TID | - | PPV OS | - |
| Mairot et al., 2022 | 2d | F | 2 | OU | HSV2 | CSF PCR | Preterm labor, positive serum PCR, negative serum IgM/IgG | Preterm (32w) | Acute respiratory failure, necrosis of temporal lobes, frontal lobes, and posterior fossa; unrecordable EEG | Posterior displacement of lens on MRI OU | Vitritis OU, optic nerve swelling OU, posterior retinitis OU | IV(Ac) 20 mg/kg TID | - | - | - |
| Kinoshita et al., 2021 | 29d | M | 2 | OU | HSV1 | Serum IgM/IgG, CSF anti-HSV antibody (viral neutralization test) | PPROM, negative serum IgM, positive serum IgG, positive serum anti-HSV antibody (viral neutralization test) | Preterm (29w), PDA, RDS | Apneic spells, rash present for 1 day, encephalomalacia | - | Iridocyclitis with posterior synechiae OU, vitritis OU, lens vacuoles OU, posterior retinitis and subretinal exudates OU, exudative RD OU → RRD OD | IV(Ac) 20 mg/kg TID x 21d + O(Ac) 150 mg/kg/day x 5m | Fluoromethalone gtts | - | 5m |
| Jia & Caiping, 2023 | 27d | M | 2 | OU | HSV2 | CSF PCR | - | Preterm (36w) | Seizures, encephalomalacia | - | Vitritis OU, disc edema OU, peripheral retinitis OU | IV(Ac) 10mg/kg TID x 21d + O(Val) 0.03g BID x 1m | - | - | 1m |
| Present case | 33d | M | 2 | OU | HSV2 | Serum PCR | Treated gonorrhea infection in pregnancy, PPROM | Preterm (30w), ROP, neonatal sepsis, IVH, RDS, PDA | - | - | Corneal haze/scar OD, vitritis OU, peripheral CRS and retinitis OU, macular scars OU | IV(Ac) 20mg/kg TID x 28d + O(Ac) 300 mg/m2 TID 6m + intravitreal foscarnet (0.72mg) OU x1 | - | Laser OU | 24m |

**Supplemental Table: Details of Individual Neonatal ARN Cases in the Literature.** Age listed is age at diagnosis of retinitis. Diagnostic methods column lists tests that yielded positive HSV results. Dosages and durations of anti-viral and steroid treatment are listed if included in the original report. *The two patients reported by Gupta et al. are twins. Abbreviations: d = day, m = month, y = year, M = male, F = female, OD = right eye, OS = left eye, OU = both eyes, HSV = herpes simplex virus, CSF = cerebrospinal fluid, PCR = polymerase chain reaction, IgM = , IgG = , PPROM = preterm premature rupture of membranes, IVF = in-vitro fertilization, di-di = dichorionic diamniotic, ROP = retinopathy of prematurity, IVH = interventricular hemorrhage, PDA = patent ductus arteriosus, RDS = respiratory distress syndrome, MRI = magnetic resonance imaging, RD = retinal detachment, RRD = rhegmatogenous retinal detachment, TRD = tractional retinal detachment, CRS = prior chorioretinal scarring, right arrows (→) denote changes in exam over time, IV = intravenous, O = oral, Ac = acyclovir, Val = valacyclovir, BID = twice daily, TID = three times daily, gtts = drops.
